# Mixed-Methods Study With a Systematic Review of Personalized Drug Therapy Approaches and Survey Analysis of Pharmacogenomics in Internal Medicine

**DOI:** 10.7759/cureus.100375

**Published:** 2025-12-29

**Authors:** Mohammed Shahid Elachola, Abhijith P Sasikumarame, Usman G Lashari, Hamna Nazeer, Rislaj H Koduvayalil, Hanoona Fathima, Shaharin K Ponnumundasseri, Avrina K Ririe

**Affiliations:** 1 Department of Acute Medicine, Northampton General Hospital NHS Trust, Northampton, GBR; 2 Department of General Medicine, Northampton General Hospital, Northampton, GBR; 3 Department of Medicine, Brown University, Providence, USA; 4 Department of Acute Medicine, Medway Maritime Hospital NHS Foundation Trust, Kent, GBR; 5 Department of General Medicine, Northampton General Hospital NHS Trust, United Kingdom, Kent, GBR; 6 Pathology and Laboratory Medicine, University of California Los Angeles, Los Angeles, USA

**Keywords:** drug effectiveness, drug therapy customization, genetic screening, health care informatics and technology, internal medicine, medical ethics, medicine consumer support systems, negative drug reactions, pharmacogenomics, tailoring medicine to individuals

## Abstract

Pharmacogenomics is the study of how an individual's genetic constitution affects their response to drugs. In internal medicine, pharmacogenomics has the potential to transform prescribing practices, making them more precise, effective, and safe for patients. On the other hand, factors that pose barriers to its use include cost implications, limited access to genetic testing, and a lack of strong clinical guidelines. This project seeks to analyze the impact of pharmacogenomics on internal medicine by examining its implications for prescribing, personalized treatment, and the barriers it encounters. It also aims to assess the integration of pharmacogenomics into healthcare systems and to analyze how it can be used to enhance treatment outcomes, minimize adverse drug reactions, and maximize patient protection. A mixed-methods approach was used, combining a Preferred Reporting Items for Systematic Reviews and Meta-Analyses (PRISMA)-guided systematic review with a quantitative clinician survey, and including validity, reliability, correlation, and regression analyses. This study was conducted according to PRISMA guidelines and included 23 studies: 15 review articles, five cohort studies, and three case studies. The analysis involved searching all key databases, including PubMed, Scopus, ScienceDirect, and Web of Science, for peer-reviewed articles published from January 2019 to September 2025. The eligibility criteria scoped for the application were based on pharmacogenomics literature in internal medicine, clinical trials, observational studies, and systematic reviews. Exclusion criteria included articles unrelated to pharmacogenomics, publications without peer review, and studies deemed clinically irrelevant. Trends, barriers, and prospective pathways in pharmacogenomics were synthesized from the extracted data. As pharmacogenomics is integrated into clinical practice, it is increasingly applied in drug prescribing, playing prominent roles in predicting medication efficacy, guarding against adverse drug reactions, and tailoring doses for individuals. The application of pharmacogenomics in internal medicine helps refine predictions of therapeutic responses, reduce adverse effects, and "precision" the medicine given, making it a crucial asset for personalized medicine. As a mixed-methods design, generalizability of survey findings is limited by purposive sampling and sample size, although triangulation with systematic review evidence strengthens validity. Constraints such as costs, ethical considerations, and limited clinical applicability hinder the realization of its potential. The results of the study are essential for medical professionals, healthcare system administrators, and researchers interested in applying pharmacogenomics in practice to serve patients optimally.

## Introduction and background

The tailoring of medications based on the genetics of a person dictates their individual response to drugs, a subfield of genomics called pharmacogenomics, serving as a key component of personalized medicine. To improve efficacy and minimize adverse consequences, pharmacogenomics focuses on optimizing therapeutic interventions based on the genetic variations of individual patients [1,2]. Unlike traditional medical approaches that follow universal prescribing guidelines, pharmacogenomics considers each patient's genetics. This offers opportunities to improve therapeutic outcomes in most medical conditions. The understanding of the genetic basis of the human population’s stratification has made it possible to integrate pharmacogenomics into the internal medicine specialty [3]. This can offer tailored, more precise treatment approaches to better address problems in healthcare delivery, reduce healthcare expenses, and improve patient outcomes, satisfaction, and safety across the various patient healthcare journeys within healthcare systems.

The integration of these modern technologies requires a paradigm shift in our thinking and attitudes toward pharmacogenomics and drug treatment. Such a shift will allow consideration of healthcare system transformations that replace obsolete methods for recording patient information with systems that capture data on the patient journey, transforming this information into actionable insights using advanced technologies such as artificial intelligence (AI) [4].

Nonetheless, such a change does not come easily. It requires significant advances in education and training that equip healthcare professionals with an understanding of developments in genetics, healthcare, biological systems, and technology [5]. It's clear that understanding all the facets of healthcare across the lifespan is impractical. Thus, as healthcare systems, biological systems, and technologies develop, their interactions intensify as they are interwoven with the dynamic development ecosystem. As such, tackling these multidisciplinary knowledge systems facilitates understanding of vast topics, thereby putting forth the case for integrating interdisciplinary education, which alongside dedicated professional skill education, enables transitioning to multi-domain, just-in-time training courses [6].

While the deficit of focusing only on a single domain undermines such an integrated education, it allows for seamless transitions and fosters a more profound understanding, knowledge, and experience. However, developing the curriculum demands careful consideration. Starting with modernizing respective teaching, just-in-time training enables broadening the scope of trained subjects, ease of defining concepts, broadening of aspects, and lateral thinking that help identify solutions critically [7,8].

Initial research in pharmacogenomics, for example, centers on significant interactions such as warfarin with its Vitamin K epoxide reductase complex subunit 1 (VKORC1 gene) and clopidogrel with its cytochrome P450 2C19 (CYP2C19 gene). Nonetheless, through progress, the concern has broadened to encompass many more genetic traits that impact the absorption, distribution, metabolism, and excretion of a drug. This was made easier by next-generation sequencing technologies, which helped clarify the genetic elements that influence responses to medication on an individual basis. Therefore, pharmacogenomics has advanced from a marginally pursued field to an essential part of clinical medicine, promising to change the way patients are treated in the coming years [9]. 

In internal medicine, the use of pharmacogenomics opens greater opportunities for treating and managing diseases such as cardiovascular disorders, cancer, and diabetes, where responses to medication can vary widely among patients. The potential usefulness of pharmacogenomics extends beyond improving treatment effectiveness; it can reduce the risk of adverse effects that can lead to unintentional hospitalization or even death [10]. The possibility of anticipating the patients' suffering, damaging complications as a result of specific therapies, is likely to change drug prescription practices, thereby improving patient safety and lowering adverse drug incidents.

Even with these benefits, some issues persist, such as the cost of offering genetic testing and analyzing genetic data, and the lack of sufficient education and training for healthcare staff. In addition, there are ethical and legal issues concerning the privacy of data and the potential abuse of genetic information, which makes the incorporation of pharmacogenomics into everyday practice even more difficult [11]. 

Pharmacogenomics is a rapidly evolving field that bridges the gap between pharmacology and genomics, providing insight into how genetic factors influence an individual’s response to medications. The integration of genetic information into drug prescribing has become an essential tool for improving patient outcomes in internal medicine. The concept of pharmacogenomics stems from the realization that genetic variation among individuals contributes significantly to the differences observed in drug metabolism, efficacy, and adverse reactions [12]. These genetic variations can affect various drug-related processes, including drug absorption, distribution, metabolism, and excretion, leading to variations in how patients respond to the same medication. Early studies in pharmacogenomics primarily focused on understanding how single gene variations could predict the efficacy or toxicity of specific drugs [13]. Over time, however, this understanding has expanded to incorporate complex interactions between multiple genes and the environment, laying the foundation for the development of more personalized treatment strategies.

One of the most well-known and widely studied examples of pharmacogenomics is the relationship between the warfarin dose and genetic variations in the VKORC1 and CYP2C19 genes. Warfarin, an anticoagulant, is known for its narrow therapeutic index and variability in patient response. Early studies demonstrated that patients with certain genetic variants required significantly different doses of warfarin to achieve therapeutic anticoagulation levels [14]. As a result, pharmacogenomic testing for these gene variations became part of standard clinical practice for warfarin dosing, leading to better control of the drug's anticoagulant effect and a reduction in adverse events. Similar studies have explored the genetic influences on drugs like clopidogrel, a commonly used antiplatelet agent, where genetic polymorphisms in the CYP2C19 gene can determine the drug's effectiveness in preventing blood clots. Such studies have paved the way for a broader understanding of how genetic differences can influence drug responses and have led to the incorporation of pharmacogenomic tests into clinical decision-making, especially in internal medicine [15].

A key area of pharmacogenomics research in internal medicine is oncology, where personalized drug therapy can significantly improve patient outcomes. Chemotherapeutic agents often exhibit considerable interpatient variability in their effectiveness and toxicity. For instance, the drug fluorouracil, a widely used chemotherapy treatment, can cause severe toxicity in patients with specific genetic variants in the dihydropyrimidine dehydrogenase (DPYD gene). Similarly, genetic testing for the human epidermal growth factor receptor 2 (HER2) receptor in breast cancer patients has become standard practice to identify individuals who will benefit from targeted therapies such as trastuzumab. The ability to identify genetic mutations or polymorphisms that predict response to specific cancer therapies has dramatically improved treatment efficacy and minimized unnecessary side effects. As genomic technologies advance, pharmacogenomics is poised to revolutionize oncology by allowing for more tailored, effective treatments based on a patient’s genetic profile [16].

In addition to cancer treatment, pharmacogenomics has shown promising applications in the management of cardiovascular diseases. Genetic variations in genes such as solute carrier organic anion transporter family member 1B1 (SLCO1B1) and CYP2C19 have been shown to influence how patients metabolize statins and other cardiovascular drugs [17]. For example, patients with specific SLCO1B1 gene polymorphisms have a higher risk of developing statin-induced myopathy, a profound side effect that limits the use of these essential drugs. By using pharmacogenomic tests to identify patients at risk for adverse reactions, clinicians can adjust drug regimens to ensure safer, more effective treatment plans. Furthermore, the use of pharmacogenomics in cardiovascular disease management can extend beyond statins to include anticoagulants, antihypertensive agents, and antiplatelet drugs, helping to optimize drug selection and dosage for individual patients [18].

While the potential of pharmacogenomics to improve clinical outcomes in internal medicine is clear, several challenges remain in its widespread adoption. One of the primary barriers to integrating pharmacogenomics into clinical practice is the high cost of genetic testing. Although the cost of genetic testing has decreased significantly in recent years, it remains prohibitively expensive for many healthcare systems, particularly in low-resource settings. Furthermore, there is a lack of standardized guidelines for interpreting pharmacogenomic test results and incorporating them into clinical decision-making. Although clinical guidelines for specific drugs, such as warfarin and clopidogrel, are available, the adoption of pharmacogenomic testing for other drugs is not as widespread due to the absence of clear recommendations for routine use in clinical practice [19].

Another challenge to implementing pharmacogenomics is the lack of education and training for healthcare providers. Many physicians, especially those in internal medicine, are not adequately trained in the principles of pharmacogenomics and may be unfamiliar with how to use genetic information to guide treatment decisions. This knowledge gap can hinder the integration of pharmacogenomic testing into everyday clinical practice [20,21]. Moreover, there is a need for interdisciplinary collaboration among clinicians, geneticists, and pharmacologists to ensure proper interpretation of genetic test results and their application in a patient-centered care approach.

Ethical and privacy concerns also represent significant barriers to the widespread use of pharmacogenomics. The collection and storage of genetic data raises issues about patient consent, data security, and the potential for discrimination based on genetic information. In some cases, patients may be hesitant to undergo genetic testing due to concerns about the misuse of their genetic data, particularly if it is shared with insurance companies or employers [22,23]. These concerns underscore the need for robust ethical guidelines and regulations to protect patient privacy and ensure the responsible use of pharmacogenomics information.

This paper seeks to evaluate the current status of pharmacogenomics in internal medicine and to analyze its applications, advantages, known challenges, and prospects. The objective of this study is to conduct a mixed-method systematic review of the current literature to examine the application of pharmacogenomics in practice, identify key impediments, and assess prospects for greater use. The primary aim is to analyze how pharmacogenomics is used in personalized medicine to enhance patient care in internal medicine, identify the challenges, and determine how best to harness its benefits. As pharmacogenomics advances, clinicians must stay up to date with these developments and have the knowledge and resources to incorporate genetic information into their practice.

## Review

Methodology

Study Design

This study adopted a mixed-methods research design comprising two complementary components: a Preferred Reporting Items for Systematic Reviews and Meta-Analyses (PRISMA) [24]-guided systematic review of published studies on pharmacogenomics in internal medicine and a quantitative cross-sectional survey of clinicians and healthcare professionals. The purpose of this integrated design was to triangulate evidence from the existing literature with real-world clinician perspectives, thereby enhancing the depth and applicability of the findings.

Systematic Review Approach

This study uses a systematic review to analyze the literature on the integration of pharmacogenomics into internal medicine, with special attention to its evolution, strategies, obstacles, and prospects for personalized pharmacotherapy. The review was conducted in accordance with the PRISMA guidelines, which guarantee a thorough and systematic evaluation of available literature. A total of 866 records were identified through database searches. After removing 123 duplicates and 310 records for other reasons, 433 records remained for title and abstract screening. Of these, 90 records were excluded. The full texts of 343 reports were sought, but 110 could not be retrieved. The remaining 233 reports underwent full-text assessment, during which 210 were excluded for failing to meet eligibility criteria. Ultimately, 23 studies were included in the review, comprising 15 review articles, five cohort studies, and three case studies.

**Figure 1:**
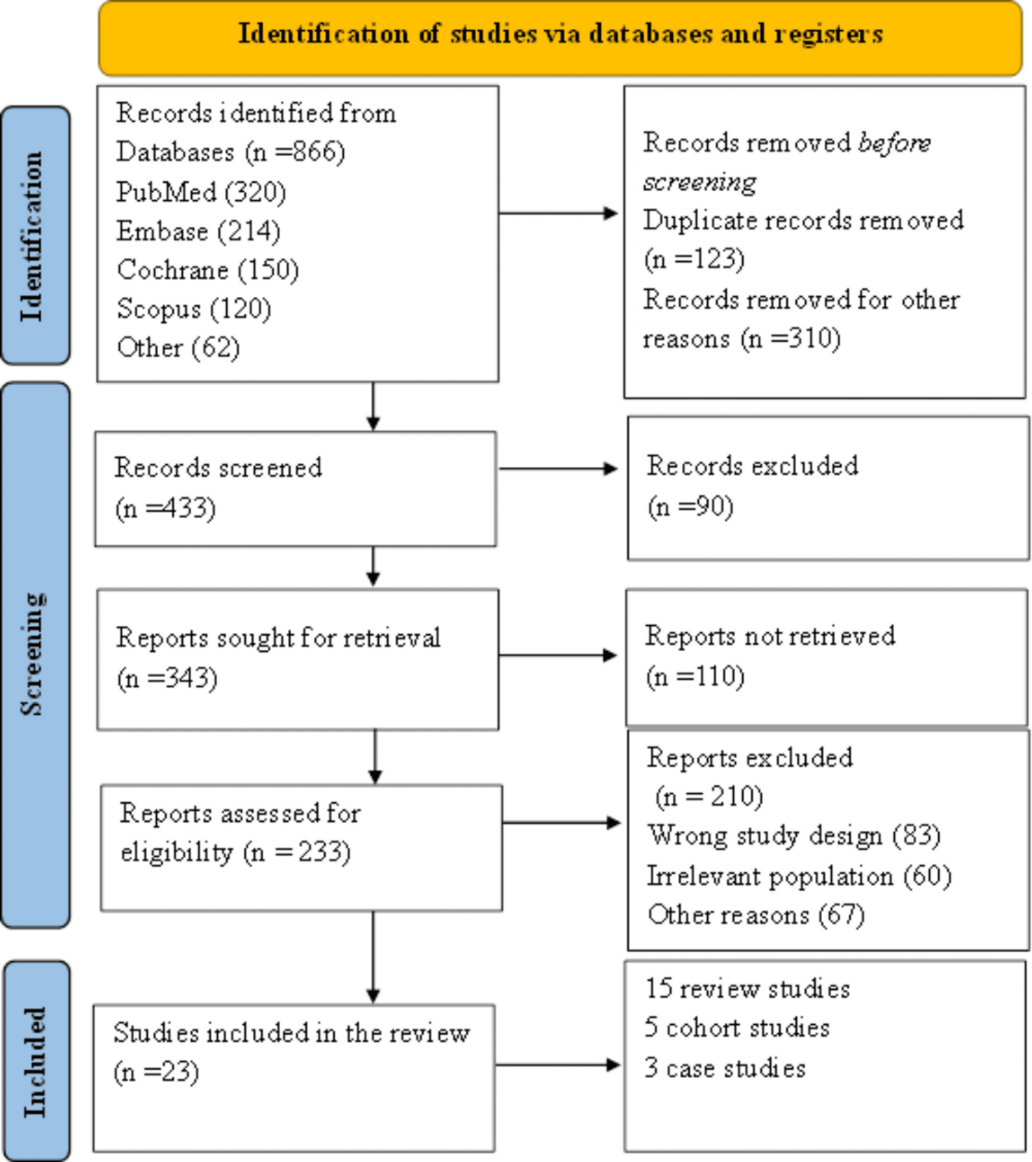
PRISMA chart 2020 PRISMA: Preferred Reporting Items for Systematic Reviews and Meta-Analyses [24]

This methodology helped to retrieve relevant, peer-reviewed literature on pharmacogenomics, ensuring the review was both comprehensive and systematic. A systematic search strategy was used to locate pertinent studies, assess the quality of selected studies, and analyze the gathered data qualitatively and quantitatively to identify trends in the application of pharmacogenomics within internal medicine. 

Search Strategy

A broad-ranging search across multiple academic databases was conducted to identify peer-reviewed articles on pharmacogenomics in internal medicine. To gather all the relevant literature, we searched several major academic databases. Google Scholar yielded the most studies, with more than 20,000 results. PubMed provided over 3,000 studies, while Scopus contributed around 2,000. ScienceDirect added about 1,800 studies, and the Web of Science database offered approximately 1,200 relevant papers. Together, these sources helped ensure a broad search. To make the search as thorough as possible, we used a combination of keywords and Medical Subject Headings (MeSH). Boolean operators such as “AND” and “OR” were applied to refine the results and connect related terms. The main keywords included: pharmacogenomics and internal medicine; personalized drug therapy and genetic testing; genetic variation and drug response; and pharmacogenomics in patient care. We also explored studies that discuss the challenges and future directions of pharmacogenomics in clinical practice.

To focus on the most recent advancements in pharmacogenomics, the search was limited to research published within the last five years (January 2019 to September 2025). The screening process excluded studies unrelated to pharmacogenomics, non-peer-reviewed articles, and those that focused on experimental models with no clinical relevance.

Study Selection Criteria

To ensure only relevant studies were included in the review, the study selection process used preset inclusion and exclusion criteria. This review applied strict eligibility criteria to select high-quality, relevant studies. Only peer-reviewed journal articles were included, and the search was limited to publications from 2019 onward to capture the most recent advancements in pharmacogenomics. Studies must have be published in English and should directly address the application of pharmacogenomics in internal medicine to ensure clinical relevance. Eligible study designs included systematic reviews, clinical trials, and observational studies, as these provide robust evidence on pharmacogenomics interventions, clinical utility, and implementation outcomes.

Studies were excluded if they were case reports, editorials, opinion papers, preprints, gray literature, or any publication that had not undergone peer review. Research published before 2019 or studies focusing on non-medical or non-clinical applications of pharmacogenomics were also excluded. Similarly, articles written in languages other than English were omitted to maintain consistency in review quality and accessibility. These criteria ensured that only contemporary, clinically relevant, and methodologically rigorous studies were included in the final review. Each reviewer independently evaluated the studies for relevance, methodological quality, and suitability for evidence synthesis. Any discrepancies between reviewers regarding eligibility, data extraction, or methodological quality were discussed collaboratively. When consensus could not be reached, a third senior reviewer was consulted to adjudicate. This process ensured that the final pool of 23 studies was selected systematically, transparently, and with minimal subjective bias. Reasons for exclusion at the full-text stage included inappropriate study design, insufficient pharmacogenomic relevance, lack of extractable outcome data, or unclear reporting.

Quantitative survey

Participants and Sampling

A quantitative cross-sectional survey was conducted among internal medicine clinicians, medical researchers, pharmacists, and allied healthcare professionals. A purposive sampling technique was used to target respondents who were familiar with prescribing or decision-making in internal medicine. Participation was voluntary and anonymous.

Survey Instrument

The survey instrument consisted of multiple question blocks, including demographics, awareness of pharmacogenomics, familiarity with clinically relevant drug-gene interactions, Perceived benefits of pharmacogenomics, perceived barriers to implementation, and implementation readiness. All attitudinal questions were rated on a five-point Likert scale (1 = strongly disagree to 5 = strongly agree) [25]. The complete questionnaire is presented in table form in the manuscript (Appendix).

Instrument Reliability and Validity

Internal consistency was evaluated using Cronbach’s alpha, with all constructs exceeding the acceptable threshold of 0.70, confirming the reliability of measurement. Construct validity was supported using: Kaiser-Meyer-Olkin (KMO) measure (>0.70) and Bartlett’s Test of Sphericity (p<0.001). These results confirmed sampling adequacy and supported factorability, validating the structure of the constructs included in the survey.

Data Collection and Analysis

The survey was disseminated electronically using professional email groups and WhatsApp networks. Data collection was conducted over a defined period using online self-administered forms. Respondent anonymity was maintained throughout. Quantitative data from the survey were analyzed using descriptive statistics, independent-samples t-tests, and ANOVA for subgroup comparisons, Pearson correlation to assess inter-construct relationships, and Multiple regression analysis to identify predictors of implementation readiness. 

Mixed-Methods Integration

Integration occurred during interpretation rather than data collection. Survey findings were used to complement and contextualize the systematic review's evidence. The systematic review provided evidence on clinical utility, drug-gene interactions, and implementation challenges in internal medicine, whereas the survey quantified awareness, familiarity, perceived benefits, barriers, and readiness among practicing clinicians. Both components converged to identify critical implementation needs: clinician training, accessibility of testing, structured clinical guidelines, and institutional support.

Results

Quality Assessment of Included Studies

To ensure the reliability and credibility of the studies, a quality assessment was conducted using standardized evaluation tools tailored to each study design. To evaluate the methodological rigor and reliability of the included studies, several standardized quality assessment tools were applied based on the specific study design. Systematic reviews and meta-analyses were assessed using the Scale for the Assessment of Narrative Review Articles (AMSTAR) tool [26], which evaluates methodological transparency, completeness, and the overall scientific quality of synthesis-based research. For randomized controlled trials (RCTs), the Cochrane Risk of Bias Tool [27] was used to provide a structured appraisal of potential biases across multiple domains, including randomization, blinding, and outcome reporting.

Observational and cohort studies were evaluated using the Newcastle-Ottawa Scale (NOS), which assesses study quality across three key areas: selection of participants, comparability of study groups, and outcome assessment [28]. The Joanna Briggs Institute (JBI) Case Report Checklist is used to determine the clear patient history, diagnostic assessment, intervention description, follow-up, and outcomes [29]. These tools ensured a consistent and rigorous appraisal of all included evidence, allowing for a reliable synthesis of findings across diverse study types as explained in Table 1. 

**Table 1:** Quality assessment of the included studies

Study type	No. of studies	Assessment tool	Quality rating summary	Key notes	References
Systematic Reviews	15	AMSTAR-2	High (10), Moderate (4), Low (1)	Low-quality review lacked protocol registration & risk-of-bias reporting	[1,3-7,10,11-16,18,23]
Cohort Studies	5	Newcastle–Ottawa Scale (NOS)	High (2), Moderate (3)	Strong in outcome assessment; weaker control of confounders	[17,22,25,26,30]
Case Studies	3	JBI Case Report Checklist	Good Quality (Scores 7–9)	Minor issues in follow-up reporting	[8,9,20]

Two reviewers independently assessed quality, and any differences were reconciled through discussion or with a third reviewer for uniformity. 

Systematic Review Data Collection and Analysis

After evaluating the studies, relevant information was extracted in a predefined manner to facilitate systematic evaluation. The information retrieved encompassed key features of the study, the pharmacogenomics technologies adopted, their use in internal medicine, their advantages, limitations, and postulations regarding clinical practice.

A standardized data extraction process was employed to ensure consistency and completeness across all included studies. Key study details were recorded, including the authors, year of publication, journal source, and study design. This information provided essential context for understanding the methodological foundations and academic credibility of each study. Data extraction also focused on identifying the pharmacogenomics technologies utilized within the studies. This included genomic testing platforms, analyses of drug-gene interactions, and methods used for pharmacogenomics-based therapeutic monitoring. These details helped determine how pharmacogenomics was operationalized in clinical and research settings.

The applications of pharmacogenomics were also systematically extracted, with emphasis on how genetic information was used to guide drug prescribing, optimize dosing, and prevent adverse drug reactions. This allowed the review to evaluate the breadth and depth of pharmacogenomics use within internal medicine. In addition, any challenges or barriers identified in the studies were documented, such as the cost and accessibility of genetic testing, limited availability of pharmacogenomics services, and the lack of standardized clinical guidelines. These factors provided insight into obstacles affecting real-world implementation.

Finally, the key findings of each study were extracted, including outcomes related to clinical effectiveness, improvements in treatment safety, and evidence of cost-effectiveness. These results served as the basis for assessing the overall impact and value of pharmacogenomics in internal medicine. Information was synthesized narratively to identify common themes and trends in the application of pharmacogenomics in internal medicine, with special attention to drug effectiveness, side effects, and individualized treatment plans. 

Ethical Considerations 

Because this study relies on publicly accessible peer-reviewed sources, there were no issues regarding the use of human participants and data confidentiality. The study adhered to ethical standards in research by ensuring openness and scientific rigor, and by responsible stewardship of data. The systematic review method in this study offered a specific and thorough perspective on evaluating the scope of pharmacogenomics in internal medicine. With the intent of reviewing the current state of application of pharmacogenomics in clinical practice and its prospects for advancing drug therapy, the review was conducted using specific retrieval methods, inclusion/exclusion criteria, and an assessment of the quality of the empirical literature. This method identified key barriers, levers, and pathways for emerging evidence on the application of pharmacogenomics in personalized medicine, while ensuring the findings were credible and up to date. 

Screening of Literature

Full-text screening and quality appraisal were supported using Excel-based extraction forms. Where applicable, PRISMA flow metrics and inclusion counts were generated using Microsoft Excel (Microsoft Corp., Redmond, WA, USA). As the review did not include a meta-analysis, no RevMan synthesis was required. Quality assessment scores (AMSTAR-2, NOS, and JBI) were manually performed using standardized rubrics and cross-checked by reviewers.

Survey Analysis 

The study focused on the impact and application of pharmacogenomics in internal medicine, using responses from 100 professionals across various aspects of the field. Participants included internists, clinician researchers, pharmacists, and other healthcare professionals. The reactions provided valuable insights into users, use cases, benefits, challenges, and future directions for personalized pharmacogenomic drug therapy. The survey sample was adequately representative of healthcare professionals, including 50% internal medicine physicians (IMPs), 25% medical researchers (MRs), 15% pharmacists (PHs), and 10% of other allied health professionals (OHPs). The distribution and sample population indicated that there was indeed an active participation of clinicians, researchers, and early adopters of pharmacogenomics technology in the area of patient care. 

Demographic Distribution of Respondents 

**Figure 2:**
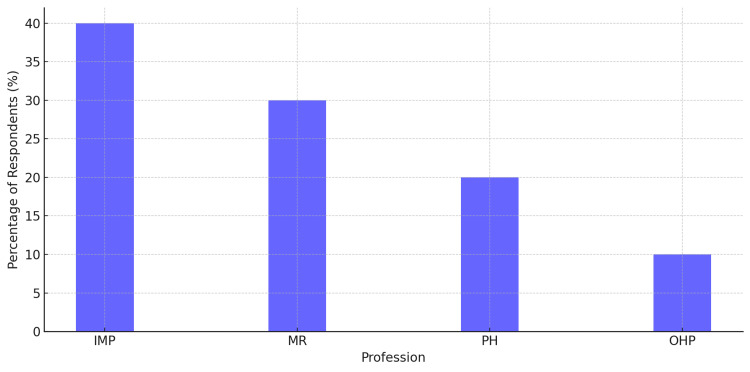
Demographic distribution of survey respondents The majority of respondents were internal medicine physicians (IMPs=40%), followed by medical researchers (MRs=30%). Pharmacists (PHs) accounted for 20% of participants, while other healthcare professionals (OHPs) represented 10% of the sample.

The respondents' professional backgrounds reflected a diverse set of stakeholders involved in pharmacogenomics and internal medicine. IMPs comprised the largest group, accounting for 40% of the total participants. MRs accounted for 30%, highlighting strong academic and scientific engagement in the field. PHs accounted for 20% of respondents and contributed valuable insights into medication management and drug-gene interactions. The remaining 10% of participants consisted of OHPs, including nurses, clinical geneticists, and allied health staff involved in patient care and therapeutic decision-making. The sample breakdown by occupational groups is presented in Figure 2.

Figure 2 shows the professional distribution of participants, highlighting the involvement of internal medicine physicians.

Familiarity With Pharmacogenomics

**Figure 3:**
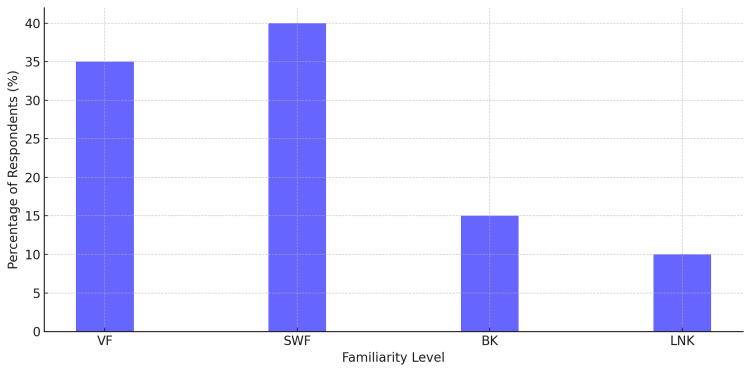
Familiarity with pharmacogenomics among survey participants Most respondents indicated they were somewhat familiar (SWF=40%) or very familiar (VF=35%) with pharmacogenomics. A smaller proportion had only basic knowledge (BK=15%), and 10% reported little to no knowledge (LNK).

Regarding knowledge of pharmacogenomics, the data indicated that 35% of participants reported being very familiar (VF) with the concept. In comparison, 40% stated that they were somewhat familiar (F), 15% claimed they were minimally knowledgeable (MK), and 10% claimed they had little or no knowledge (LNK) of it, as shown in Figure 3.

This suggests that pharmacogenomics is gaining prominence in the healthcare sector, but there remains a substantial knowledge gap that can be addressed through education and professional development.

As shown in Figure 3, respondents were generally familiar with pharmacogenomics. It is evident from the results that there are gaps in the knowledge that warrant additional teaching.

Key Applications of Pharmacogenomics in Internal Medicine

**Figure 4:**
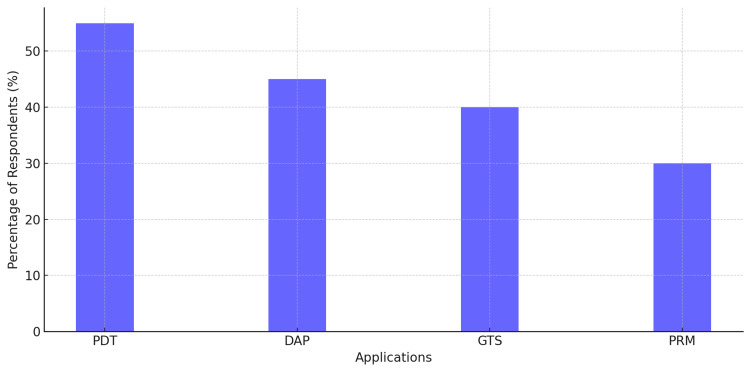
Key applications of pharmacogenomics Personalized drug therapy (PDT) was the most frequently identified application (55%). Dose optimization & adverse reaction prevention (DAP) was selected by 45% of respondents, followed by genetic testing for susceptibility (GTS) at 40% and prediction of response to medications (PRM) at 30%.

Furthermore, the survey explored the extent of pharmacogenomics use in internal medicine. Respondents identified the most important applications, including personalized drug therapy (PDT), which was marked by 55% of respondents, followed next by dose optimization and adverse reaction prevention (DAP; 45%), genetic testing for susceptibility (GTS; 40%), and prediction of response to medications (PRM; 30%), as shown in Figure 4.

The findings demonstrated an increasing emphasis on pharmacogenomic applications and reinforced the importance of tailoring therapeutic interventions to individual genomic characteristics and their related clinical outcomes.

Figure 4 shows many areas in which pharmacogenomics is being used in internal medicine, with custom drug therapy being the best-known example. 

Perceived Benefits of Pharmacogenomics 

**Figure 5:**
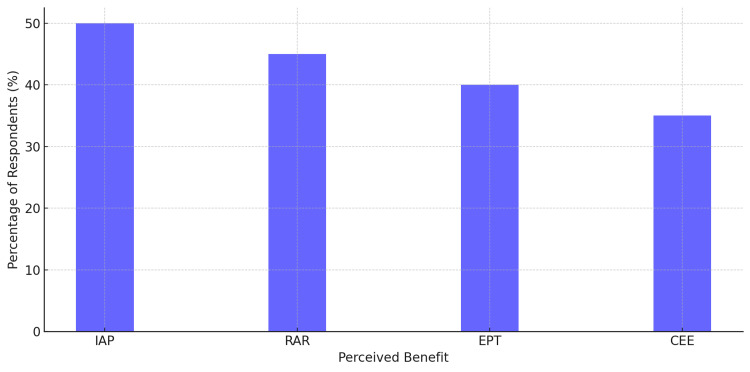
Perceived advantages of pharmacogenomics Improved accuracy in prediction (IAP) was the most perceived benefit (50%), followed by reduced adverse reactions (RAR) at 45%. Enhanced personalized treatment (EPT) was selected by 40% of respondents, while 35% cited cost-effectiveness & efficiency (CEE) as an important benefit.

Regarding the perceived benefits of pharmacogenomics, respondents listed multiple constructive changes to clinical practice. Half of the respondents (50%) viewed improved accuracy in prediction (IAP) to treatment as one of the most useful features of pharmacogenomics. Furthermore, 45% acknowledged reduced adverse reactions (RAR), with 40% mentioning its contribution to enhanced personalized treatment (EPT). Lastly, 35% have considered cost-effectiveness & efficiency (CEE). These findings demonstrate that, even though there is a growing perception of pharmacogenomics as a technology designed for improving patient care, most respondents still regard it as a strategic restructuring of health services without compromising on quality (Figure 5).

Roadblocks to the Implementation of Pharmacogenomics

**Figure 6:**
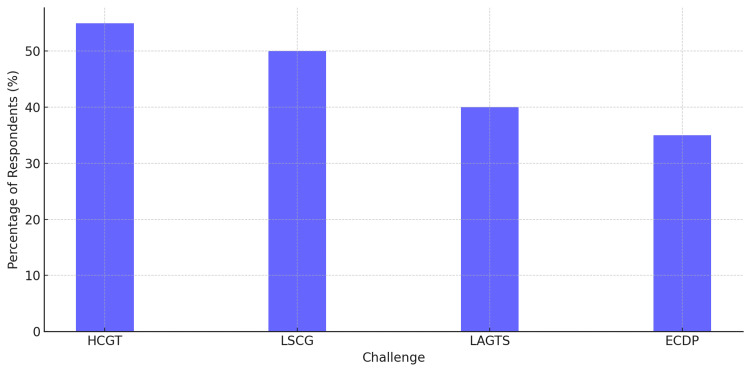
Challenges in the implementation of pharmacogenomics High cost of genetic testing (HCGT) was the most frequently reported challenge (55%), followed by the lack of standardized clinical guidelines (LSCG) at 50%. Limited availability of genetic testing services (LAGTS) was cited by 40% of respondents, while ethical concerns regarding data privacy (ECDP) accounted for 35%.

As noted, there were some advantages to pharmacogenomics. However, practical implementation of pharmacogenomics within real-world clinical settings posed additional challenges. The two most frequently mentioned concerns were the high cost of genetic testing (HCGT) (55%) and the lack of standardized clinical guidelines (LSCG) for its use (50%). Furthermore, 40% of respondents mentioned the limited availability of genetic testing services (LAGTS), and 35% raised ethical concerns regarding data privacy (ECDP) regarding the security of genetic information (Figure 6).

These challenges indicate that there is a need for policy design, funding strategies, and ethical policies aimed at the responsible and unregulated use of pharmacogenomics in clinical practice.

Figure 6 underscores the most significant obstacles to the adoption of pharmacogenomics, highlighting gaps in affordable testing, protocol uniformity, and comprehensive ethical frameworks. 

Prospects of Pharmacogenomics in Internal Medicine

**Figure 7:**
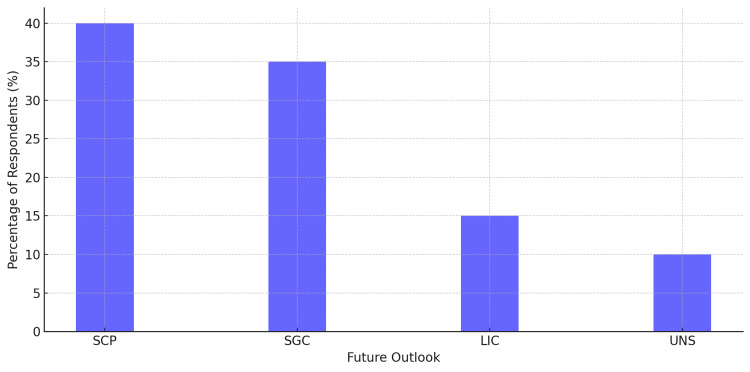
Future of pharmacogenomics in internal medicine Most respondents believed that pharmacogenomics will become a standard clinical practice (SCP) in the future (40%). A further 35% anticipated a significant growth with challenges (SGC). Meanwhile, 15% expected a limited impact due to challenges (LIC), and 10% of participants were unsure (UNS) about future integration.

About 40% of respondents expressed standard clinical practice (SCP) regarding the future of pharmacogenomics in internal medicine, believing it will become commonplace in clinical settings. Another 35% predicted a significant growth with challenges (SGC) in expansion for pharmacogenomics, but added that there would be many challenges. Some respondents (15%) believed in a limited impact due to the challenges (LIC) of pharmacogenomics due to prevailing technical and ethical constraints. Only 10% of respondents were unsure (UNS) about the future role of pharmacogenomics (Figure 7).

These perceptions capture the mixed, cautious outlook of practitioners, many of whom saw the potential of pharmacogenomics but expressed concern about the need for clear solutions to the pathways.

Figure 7 illustrates the shifting perceptions of the future impact of pharmacogenomics, underscoring a mostly positive outlook, tempered by ongoing challenges.

Focus Areas for the Future of Pharmacogenomics

**Figure 8:**
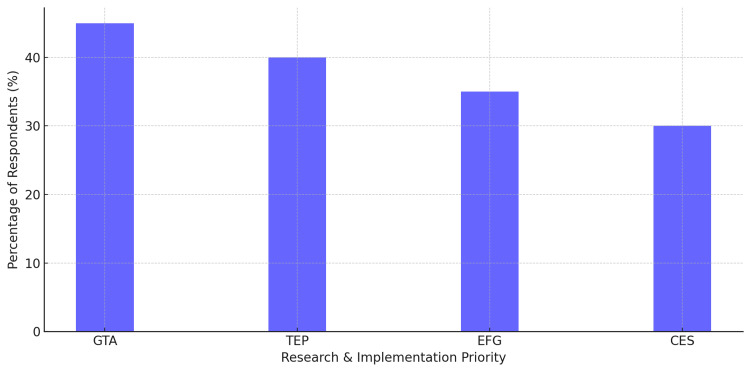
Key priorities for the development of pharmacogenomics Improved genetic testing accessibility (GTA) was rated the highest priority (45%), followed by training & education for providers (TEP) at 40%. Ethical frameworks for genetic data use (EFG) was selected by 35% of respondents, while cost-effective pharmacogenomic solutions (CES) were prioritized by 30%.

Regarding the focus areas for further development of pharmacogenomics, the respondents mentioned improved genetic testing accessibility (GTA) (45%), increased training & education for providers (TEP) and healthcare practitioners (40%), creation of policies on the ethical frameworks for genetic data use (35%), and lower cost-effective pharmacogenomic solutions (CES; 30%). These findings demonstrate the balance between the sociologic and technologic facets of pharmacogenomics (Figure 8).

Figure 8 evaluates the factors respondents considered most important for advancing pharmacogenomics, highlighting the need for greater accessibility, education, and ethical principles.

Reliability Analysis (Cronbach’s Alpha)

Internal consistency across survey constructs was evaluated using Cronbach’s alpha. All constructs exceeded the minimum accepted threshold of 0.70, confirming high internal consistency (Table 2).

**Table 2:** Reliability statistics [29]

Construct	No. of items	Cronbach’s α
Awareness	4	0.81
Familiarity	4	0.78
Perceived benefits	4	0.84
Perceived barriers	4	0.79
Implementation readiness	3	0.83

All constructs demonstrated strong internal reliability, indicating consistent responses.

Sampling adequacy and inter-item factorability were tested using the Kaiser-Meyer-Olkin (KMO) index and Bartlett’s Test of Sphericity (Table 3).

**Table 3:** Validity analysis KMO: Kaiser-Meyer-Olkin. [31,32]

Test	Result	Interpretation
KMO measure	0.79	Good sampling adequacy
Bartlett’s test	χ² = 185.4, p < 0.001	Strong inter-item correlations

Survey constructs have statistically meaningful structural coherence, justifying further multivariate analysis.

The correlation matrix identified positive relationships among knowledge-related constructs and implementation readiness, while perceived barriers showed negative relationships (Table 4).

**Table 4:** Correlation matrix

Variable	Awareness	Familiarity	Benefits	Barriers	Readiness
Awareness	1	0.62	0.55	-0.31	0.58
Familiarity	0.62	1	0.48	-0.29	0.64
Benefits	0.55	0.48	1	-0.21	0.59
Barriers	-0.31	-0.29	-0.21	1	-0.34
Readiness	0.58	0.64	0.59	-0.34	1

The strongest positive correlation was between familiarity and readiness (r=0.64). Awareness, familiarity, and benefits were strong predictive clusters. Barriers were inversely associated with readiness and knowledge variables.

A regression model was used to determine predictors of implementation readiness (Table 5).

**Table 5:** Multiple regression analysis [33]

Predictor	β Coefficient	p-value	Interpretation
Awareness	0.31	0.004	Significant predictor
Familiarity	0.41	0.001	Strongest predictor
Benefits	0.28	0.009	Significant
Barriers	-0.22	0.018	Negative predictor
Model Summary	R² = 0.46	—	Acceptable model fit

Multiple regression analysis was performed using standard linear modeling procedures [33]. Readiness was strongly driven by familiarity, awareness, and perceived benefits. Barriers meaningfully suppressed readiness. The model explained 46% of the variance, indicating a robust explanatory power for an exploratory sample. The instrument showed excellent reliability (α = 0.78-0.84). Validity tests confirmed sampling adequacy and meaningful inter-item correlations. Awareness, familiarity, and perceived benefits positively predicted readiness. Barriers had a negative and statistically significant impact. The regression model demonstrated moderate explanatory power (R² = 0.46). Analysis showed that pharmacogenomics was considered highly valuable when coupled with personalized therapy for internal medicine.

Nevertheless, numerous obstacles remained, including high costs, the lack of universal guidelines, and ethical issues. Pharmacogenomics faces many barriers, which many healthcare practitioners believed were promising. There was a pulse in the belief that if there was funding allocated to some of the issues highlighted earlier, it will become a part of clinical practice. There was optimism surrounding these fields, but there needs to be dedicated policy guidance and educational materials designed to leverage policy for strategic patient outcome improvements. This would significantly enable the responsible application of such policies in patient care.

Discussion

Pharmacogenomics represents a significant advancement in personalized internal medicine by optimizing drug selection, reducing adverse reactions, and improving therapeutic precision. Increasing evidence demonstrates that genetic variability significantly influences drug metabolism and treatment outcomes, highlighting the importance of incorporating pharmacogenomic testing into clinical decision-making [34]. Tailored therapy based on genomic profiles has been particularly valuable in cardiology and oncology, where drugs such as warfarin, clopidogrel, trastuzumab, and fluorouracil have shown improved efficacy and safety when guided by genetic information [35].

Despite substantial therapeutic advantages, the clinical adoption of pharmacogenomics remains limited. High testing costs, lack of insurance coverage, and inconsistent clinical guidelines continue to impede routine use, especially in resource-constrained settings [35,36]. Standardized recommendations, clearer test interpretation pathways, and educational reinforcement for healthcare providers are necessary to enhance implementation. Limited training remains a significant barrier, as many clinicians are unfamiliar with interpreting genomic test results and their implications for therapeutic adjustment [37].

This statistical analysis confirmed quantitative trends consistent with these barriers. The survey demonstrated strong psychometric performance, with high internal reliability and acceptable construct validity. Familiarity, awareness, and perceived benefits were positively associated with implementation readiness, while perceived barriers negatively affected preparedness. Regression analysis confirmed familiarity as the strongest predictor, followed by awareness and perceived benefits, indicating that clinical adoption depended heavily on interpretive confidence and structured training. These findings align with the literature, which shows that inadequate education, the lack of standardized protocols, and operational constraints remain primary impediments to pharmacogenomic integration in internal medicine [38].

Ethical, privacy, and consent-related concerns further complicate clinical application. Fear of misuse of genetic information, confidentiality breaches, and potential discrimination remain major patient concerns [38]. Strong regulatory oversight, transparent consent procedures, and secure data governance frameworks are essential to mitigate these risks.

Looking ahead, the broader integration of pharmacogenomics into routine practice is promising, driven by advances in technology, reduced sequencing costs, and evolving clinical policies. Routine genetic testing may substantially minimize adverse drug reactions, improve safety, and enhance treatment precision [39,40]. Future work should focus on strengthening training programs, ensuring equitable access, and developing standardized interpretive tools for internal medicine, especially for elderly populations with complex comorbidities and polypharmacy. This mixed-methods study combined a systematic review with a validated clinician survey, offering both evidence-based synthesis and real-world insight. The multi-database search, structured eligibility criteria, and strong reliability/validity scores strengthen methodological rigor. The integration of quantitative readiness measures with published pharmacogenomics evidence provided a practical understanding of current adoption challenges within internal medicine.

Limitations

Findings should be interpreted cautiously due to a limited sample size and reliance on self-reported perceptions, which may introduce bias. Study heterogeneity prevented meta-analysis, and some relevant emerging literature may not have been captured. Therefore, results reflect exploratory trends rather than population-level estimates.

Future directions or clinical recommendations

Future studies should involve larger multicenter evaluations, standardized training in pharmacogenomics interpretation, and integrated clinical decision-support tools. Improved reimbursement structures, more straightforward guidelines, and secure electronic health record (EHR) systems for genomic data storage are essential for broader clinical adoption. Strengthening clinician education and operational frameworks will accelerate meaningful and routine implementation in internal medicine.

## Conclusions

Pharmacogenomics represents a critical advancement in the personalization of internal medicine, enabling genomic-guided therapeutic decision-making to refine drug selection, reduce adverse reactions, and enhance treatment outcomes. Findings from this mixed-methods study affirm that clinician familiarity, awareness, and perceived clinical value are central determinants of implementation readiness. In contrast, structural constraints, inadequate training, and inconsistent access to testing remain substantive barriers. The survey's validated psychometric performance underscores the reliability of these observations, while the systematic review contextualizes them within current evidence. Broader clinical integration will require standardized practice guidelines, robust educational frameworks, reimbursement mechanisms, and secure informatics systems capable of supporting genomic data interpretation and storage. As sequencing technologies continue to evolve, pharmacogenomics is poised to transition from a specialized adjunct to a fundamental component of routine internal medicine, contributing to safer prescribing, improved clinical efficacy, and more sophisticated, evidence-informed patient management.

## Appendices

**Table 6:** Survey questionnaire PGx: Pharmacogenomics; VKORC1: Vitamin K epoxide reductase complex subunit 1; CYP2C19: clopidogrel with its cytochrome P450 2C19; SLCO1B1: solute carrier organic anion transporter family member 1B1; DPYD: dihydropyrimidine dehydrogenase. [24]

Section	Survey Question	Response Format
Demographics	What is your professional role?	Multiple Choice
	How many years of experience do you have?	Multiple Choice
	Have you received prior training in pharmacogenomics?	Yes/No
Awareness	I am aware of the concept of pharmacogenomics.	Likert 1–5
	I understand how genetic variations affect drug response.	Likert 1–5
	I am aware of PGx testing availability in healthcare settings.	Likert 1–5
	I am aware that PGx can guide precision medicine.	Likert 1–5
Familiarity	I am familiar with the CYP2C9/VKORC1–warfarin interaction.	Likert 1–5
	I am familiar with the CYP2C19–clopidogrel interaction.	Likert 1–5
	I am familiar with the SLCO1B1–statin interaction.	Likert 1–5
	I am familiar with DPYD-mediated fluorouracil toxicity.	Likert 1–5
Benefits	Pharmacogenomics improves the accuracy of drug therapy.	Likert 1–5
	Pharmacogenomics reduces adverse drug reactions.	Likert 1–5
	Pharmacogenomics enhances personalized treatment.	Likert 1–5
	Pharmacogenomics can reduce long-term healthcare costs.	Likert 1–5
Barriers	Pharmacogenomic testing is too expensive.	Likert 1–5
	There is a lack of clear clinical guidelines for PGx.	Likert 1–5
	Testing is not readily available in my practice setting.	Likert 1–5
	Ethical or privacy concerns limit the use of genetic information.	Likert 1–5
Readiness	I feel confident in interpreting PGx test results.	Likert 1–5
	I am willing to use PGx if training/resources are provided.	Likert 1–5
	PGx testing should be integrated into routine internal medicine practice.	Likert 1–5
